# Targeting Translation Termination Machinery with Antisense Oligonucleotides for Diseases Caused by Nonsense Mutations

**DOI:** 10.1089/nat.2019.0779

**Published:** 2019-08-06

**Authors:** Lulu Huang, Mariam Aghajan, Tianna Quesenberry, Audrey Low, Susan F. Murray, Brett P. Monia, Shuling Guo

**Affiliations:** Ionis Pharmaceuticals, Carlsbad, California.

**Keywords:** translational read-through, nonsense mutation, therapeutic, antisense oligonucelotides

## Abstract

Efforts to develop treatments for diseases caused by nonsense mutations have focused on identification of small molecules that promote translational read-through of messenger RNAs (mRNAs) harboring nonsense stop codons to produce full-length proteins. However, to date, no small molecule read-through drug has received FDA approval, probably because of a lack of balance between efficacy and safety. Depletion of translation termination factors eukaryotic release factor (*eRF*) 1 and *eRF3a* in cells was shown to promote translational read-through of a luciferase reporter gene harboring a nonsense mutation. In this study, we identified antisense oligonucleotides (ASOs) targeting translation termination factors and determined if ASO-mediated depletion of these factors could be a potentially effective and safe therapeutic approach for diseases caused by nonsense mutations. We found that ASO-mediated reduction of either *eRF1* or *eRF3a* to 30%–40% of normal levels in the mouse liver is well tolerated. Hemophilia mice that express a mutant allele of human coagulation factor IX (FIX) containing nonsense mutation R338X were treated with *eRF1*- or *eRF3a*-ASO. We found that although *eRF1*- or *eRF3a*-ASO alone only elicited a moderate read-through effect on *hFIX-R338X* mRNA, both worked in synergy with geneticin, a small molecule read-through drug, demonstrating significantly increased production of functional full-length hFIX protein to levels that would rescue disease phenotypes in these mice. Overall our results indicate that modulating the translation termination pathway in the liver by ASOs may provide a novel approach to improving the efficacy of small molecule read-through drugs to treat human genetic diseases caused by nonsense mutations.

## Introduction

Genetic diseases affect ∼30 million people in the United States and ∼300 million people worldwide (http://rarediseases.info.nih.gov). An extensive meta-analysis study using the Human Gene Mutation Database has revealed that 11% of all genetic disease-causing mutations are nonsense mutations that generate translation premature termination codons (PTCs) [1]. These mutations commonly inactivate gene function because of the production of truncated proteins and may also lead to reduced messenger RNA (mRNA) levels because of rapid mRNA turnover by the nonsense-mediated mRNA decay (NMD) pathway [2–5]. Efforts to develop treatments for these diseases have focused on developing small molecule translational read-through drugs that induce the translation machinery to record a PTC as a sense codon such that translation continues in the correct reading frame to complete the synthesis of a full-length, potentially functional protein [6–8].

Translation has been shown to be a tightly regulated, high-fidelity process [9,10]. Translation termination occurs when the ribosome reads a stop codon and recruits translation termination factors, instead of aminoacyl-transfer RNAs (tRNAs), to the A-site of the ribosome. Occasionally, a near-cognate aminoacyl-tRNA is recruited to the stop codon that leads to translational read-through [6–8]. The basal level of translational read-through for a normal stop codon occurs at a frequency of 0.001%–0.1% of mRNAs [11]; however, the read-through at nonsense mutations is 10 times higher and occurs in 0.01%–1% of mRNAs [12–14]. These differences in basal translational read-through frequencies indicate that premature translation termination is different, and possibly less regulated, compared with the normal translation termination process, suggesting that pharmacological approaches may be effective when applied to harness translation termination at PTCs to further enhance read-through efficiency.

In fact, it has been demonstrated that small molecules, such as aminoglycosides and Ataluren, are able to further promote translational read-through at PTCs without affecting translation termination at normal stop codons [7,8,15]. The most utilized read-through compounds, aminoglycosides and their derivatives, have been tested in preclinical and clinical studies, demonstrating varying levels of efficacy in promoting translational read-through. However, the ototoxicity, nephrotoxicity, and retinal toxicity of aminoglycosides hamper chronic use of these drugs to treat genetic diseases caused by nonsense mutations [6–8,14,16]. Several studies using *in vitro* and *in vivo* systems provided strong evidence that these deleterious effects are not caused by their ability to bind cytoplasmic ribosomal RNA (rRNA), which promotes translational read-through, but rather by their ability to also bind mitochondrial rRNA, resulting in inhibition of mitochondrial protein synthesis. In fact, aminoglycosides have a higher affinity for mitochondrial rRNA than cytoplasmic rRNA and consequently inhibit mitochondrial protein synthesis at concentrations that promote cytoplasmic translational read-through [16–19]. Therefore, safer approaches to promoting cytoplasmic translational read-through of mRNAs harboring PTCs are desperately needed.

In eukaryotes, two translation termination factors are required for cytoplasmic translation termination, eukaryotic release factor (eRF) 1 and eRF3. eRF1 recognizes all three stop codons (UAA, UAG, and UGA) through direct interaction at the ribosome decoding A site and activates the peptidyl transferase center to trigger hydrolysis of the peptidyl-tRNA, releasing the newly synthesized polypeptide [20,21]. Translational read-through efficiency depends on competition between stop codon recognition by eRF1 and decoding of the stop codon by a near-cognate tRNA [7,8]. In fact, it has been shown that small interfering RNA (siRNA) or antisense oligonucleotides (ASOs) targeting *eRF1* promote translational read-through of a luciferase reporter in HEK293 cells [22]. eRF3 is a GTPase that binds to and stabilizes eRF1, assisting with stop codon recognition by eRF1 [23]. eRF3 also binds to Poly(A)-binding protein to stimulate translation termination and facilitates ribosome recycling [24]. Mammals have two *eRF3* genes, *eRF3a* and *eRF3b*. *eRF3b* is mostly expressed in the brain, whereas *eRF3a* is ubiquitously expressed [25]. An siRNA targeting *eRF3a*, which also leads to downregulation of eRF1 at the protein level, promotes translational read-through of a luciferase reporter in HEK293 cells [25].

These *in vitro* reporter assay results suggest that reduction of translation termination factors could be an approach to promote translational read-through at PTCs. It remains to be tested, however, if we can safely reduce the expression of translation termination factors to promote translational read-through at PTCs without perturbing translation termination at normal stop codons. Furthermore, it is unknown whether these approaches will lead to translational read-through in animals and whether the level of read-through is sufficient to ameliorate disease phenotypes. Therefore, we sought to determine if reduced levels of translation termination factors are tolerated in animals, and whether increased read-through will produce sufficient functional protein to rescue the disease phenotype in a hemophilia mouse model with nonsense mutations.

Antisense technology is a clinically validated platform for drug discovery. Ionis ASOs are short chemically modified oligonucleotides that bind specifically to their RNA targets through Watson–Crick base pairing and trigger endogenous RNase H1-mediated degradation of their targeted mRNAs [26,27]. ASOs have proven to be a specific, potent, and well-tolerated therapeutic approach for cardiovascular, metabolic, neurological, and severe genetic diseases, as well as cancer [28]. In this study, we initiated an effort to use ASOs to target the translation termination factors to determine if ASO-mediated reduction of *eRF1* or *eRF3a* could be a potentially effective and safe therapeutic approach for diseases caused by a nonsense mutation.

## Materials and Methods

### Antisense oligonucleotides

ASOs used in this study were chemically modified with phosphorothioate in the backbone and 2′-methoxyethyl (MOE) modification in the wings with a central 10-nucleotide deoxy gap (5-10-5 gapmer). Oligonucleotides were synthesized using an Applied Biosystems 380B automated DNA synthesizer (PerkinElmer Life and Analytical Sciences) and purified as previously described [27,29]. Lyophilized ASOs were dissolved in sterile Dulbecco's phosphate-buffered saline (DPBS; without calcium or magnesium) and quantified by ultraviolet spectrometry, diluted to the desired concentration, sterilized through a 0.2 μm filter.

### Animals

All the animals of wild-type (C57BL/6; purchased from JAX) and hemophilia mice [30] (licensed from The Children's Hospital of Philadelphia and maintained in Taconic) were housed under standard condition in a pathogen-free mouse facility. All animal procedures were performed in accordance with National Institution of Health guidelines and approved by the Institutional Animal Care and Use Committee at Ionis Pharmaceuticals. ASOs were administrated subcutaneously at a volume of 10 μL/g. Geneticin (G418; 5.6 mg/mL) was administrated subcutaneously at a volume 5 μL/g.

### Plasma chemistry analysis

Blood samples were collected by cardiac puncture at time of sacrifice. Plasma chemistry values were measured on the AU480 Clinical Chemistry Analyzer (Beckman Coulter).

### RNA analysis

Animal tissues were homogenized in guanidine isothiocyanate solution (Invitrogen) supplemented with 8% 2-mercaptoethanol (Sigma-Aldrich). Total RNA was prepared using the RNeasy Mini Kit (Qiagen). Quantitative real-time polymerase chain reaction (qRT-PCR) was performed using Express One-Step Superscript qRT-PCR kits on an ABI StepOne Plus Real-Time PCR system. Taqman primer probe sets: *eRF1* forward ATTTCCAGGGAATGGAGTACCA, reverse GGGTATGCTCCTTGGGTTGA, probe CGGCAACCGTGCCTCACCCT; *eRF3a* forward CGGAACCTGTAGAGTCCTCTCAA, reverse CATTTCTGTCTCTCCATTTTCTACAACA, probe TCGTGTGAAGGTTCAAA; *hFIX* forward TGAGGAAGAATTCAACAGTGTGTCT, reverse CCTCTGGTCTAGGCAACTTCAAC, probe CAGCAGTGTTCAGAGCCAAGCAAGA; C/EBP homologous protein (*CHOP*) forward TGAGCCTAACACGTCGATTATATCA, reverse TCTGGAACACTCTCTCCTCAGGTT, probe CAGCGACAGAGCCAGAATAACAGCCG; glyceradehyde 3-phosphate dehydrogenase (*Gapdh*) forward GGCAAATTCAACGGCACAGT, reverse GGGTCTCGCTCCTGGAAGAT, probe AAGGCCGAGAATGGGAAGCTTGTCATC.

### Protein analysis

Protein levels were measured using western blot. Animal tissues were homogenized in RIPA buffer (Thermo Fisher Scientific) containing Halt Protease Inhibitor Cocktail (Life Technologies). Protein concentrations were determined using the BioRad DC protein assay, and protein was loaded at 40 μg onto a 4%–15% Criterion™ TGX™ Precast Midi Protein Gel (BioRad). Western blot membranes were probed with primary anti-eRF1 antibody (ab31799; Abcam) or anti-eRF3a antibody (ab49878; Abcam). An antibody against β-actin (A5316; Sigma) was used as a loading control. Membranes were then incubated with IRDye secondary antibodies (Li-COR) and scanned using an Odyssey infrared system (Li-COR). Images were quantified using Image Studio (Li-COR).

hFIX protein level in mouse plasma was measured by enzyme-linked immunosorbent assay (ELISA) using Human Factor IX (FIX) ELISA Kit (ab188393; Abcam) following manufacture instructions.

### FIX activity assay

FIX activity assay was performed at UCSD Murine Hematology and Coagulation Core Laboratory. In brief, clotting times were determined in duplicate with an ST4 semi-automated mechanical coagulation instrument (Diagnostica Stago). Thirty microliters of citrated sample plasma diluted 1/10 in HN/BSA buffer was incubated with 30 μL of APTT reagent and 30 μL of human citrated plasma deficient of FIX at 37°C for 5 min, followed by the addition of 30 μL of 25 mM 37°C CaCl_2_ to initiate clotting. Time until clot formation was measured and interpolated on a standard curve of serial dilutions citrated normal (BL/6 pool) mouse plasma tested as described. Final result was reported in % *hFIX-WT* mice.

## Results

### ASO-mediated reduction of *eRF1* and *eRF3a* is well tolerated in mice

**FIG. 1.**
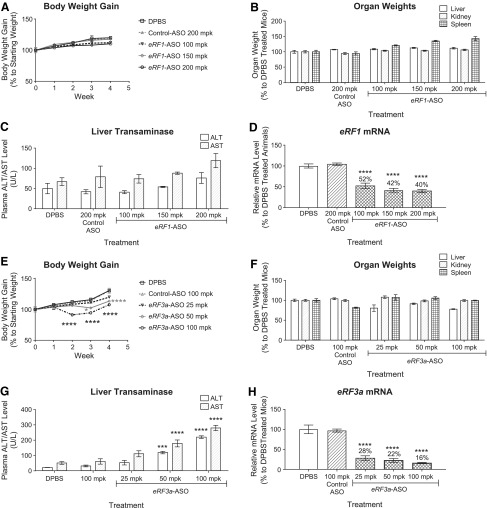
Reduction of *eRF1* to ∼40% and *eRF3a* to ∼30% of normal levels in mouse liver is well tolerated. **(A–D)** Male C57BL/6 mice 7 weeks of age (*n* = 4) were treated with *eRF1*-ASO at 100, 150, or 200 mg/kg/week. DPBS and a scrambled ASO dosed at 200 mg/kg/week were used as controls. Animals were dosed twice a week for a total of eight doses in a 4-week period. Necropsy was performed 48 h after the last dose of ASO. Results are presented as mean ± standard error. **(A)** Body weights measured once a week. **(B)** Liver, kidney, and spleen weights measured at necropsy. **(C)** Plasma ALT and AST levels measured by clinical analyzer at the time of necropsy. **(D)** qPCR analysis of *eRF1* mRNA levels in mouse liver samples. Mouse *Gapdh* mRNA was used as endogenous control. *eRF1* mRNA level in DPBS-treated animals was set as 100%. **(E–H)** Male C57BL/6 mice 7 weeks of age (*n* = 4) were treated with *eRF3a*-ASO at 25, 50, or 100 mg/kg/week. DPBS and a scrambled ASO dosed at 100 mg/kg/week were used as controls. Animals were dosed twice a week for a total of eight doses in a 4-week period. Necropsy was performed 48 h after the last dose of ASO. Results are presented as mean ± standard error. **(E)** Body weights measured once a week. **(F)** Liver, kidney, and spleen weights measured at necropsy. **(G)** Plasma ALT and AST levels measured by clinical analyzer at the time of necropsy. **(H)** qPCR analysis of *eRF3a* mRNA levels in mouse liver samples. Mouse *Gapdh* mRNA was used as endogenous control. *eRF3a* mRNA level in DPBS-treated animals was set as 100%. Statistical significance was determined using a one-way ANOVA and Dunnett's multiple comparison test in Prism. All groups were compared with DPBS-treated group. ***P < 0.001; ****P < 0.0001. mpk, mg/kg/week; ALT, alanine transaminase; AST, aspartate transaminase; eRF, eukaryotic release factor; mRNA, messenger RNA; qPCR quantitative polymerase chain reaction; DPBS, Dulbecco's phosphate buffered saline; ANOVA, analysis of variance.

We first designed and tested ∼350 ASOs targeting mouse *eRF1* and *eRF3a* mRNAs in mouse cell lines (data not shown). The top 15 ASOs that achieved the best target reduction for each of the translation termination factors were then evaluated for *in vivo* tolerability at a single-dose level in mice (data not shown). The best tolerated ASOs were selected for subsequent experiments. To determine the threshold of translation termination factor reduction that is well tolerated in animals, we performed ASO dose–response experiments in C57Bl/6J mice. ASOs targeting mouse *eRF1* or *eRF3a* were dosed twice a week for 4 weeks at the indicated doses. Weekly body weights and also terminal organ weights and blood chemistry were measured to determine if the ASO treatments were tolerated. As liver is the most sensitive organ for ASO-mediated target knockdown with second-generation ASO chemistry, liver RNA was extracted and measured for target reduction and plasma levels of the liver enzymes; alanine transaminase (ALT) and aspartate transaminase (AST) were measured to monitor liver injury. Our results demonstrated that reduction of *eRF1* to ∼40% of normal levels in mouse liver with 200 mg/kg/week ASO treatment is well tolerated. ASO-treated mice showed normal body weight gain, organ weights, and liver transaminase levels compared with the DPBS-treated control group (Fig. 1A–D). It is possible that reduction of *eRF1* to levels <40% of normal levels could also be well tolerated; however, we could not achieve further target reduction with ASOs targeting *eRF1* (data not shown). Moreover, we observed that reduction of *eRF3a* to ∼28% of normal levels in mouse liver with 25 mg/kg/week ASO treatment is well tolerated (Fig. 1E–H). Animals exhibited normal body weight gain, organ weights, and liver transaminase levels compared with the DPBS-treated control group (Fig. 1E–H). However, when *eRF3a* was depleted to ∼22% or 16% of control levels with either 50 or 100 mg/kg/week ASO treatments, respectively, the mice showed less body weight gain and elevated plasma transaminase levels (Fig. 1E–H), indicating that reducing *eRF3a* to <28% of the normal levels in mouse liver is not well-tolerated. These results were confirmed using two ASOs for each translation termination factor (Fig. 1; data not shown).

### ASO targeting *eRF3a* moderately improves translational read-through in *hFIX-R338X* mice

It has been reported that siRNA-mediated depletion of *eRF3a* also reduces the level of eRF1 protein by affecting its stability [25]; therefore, we postulated that *eRF3a* could be a more effective target to promote translational read-through. To evaluate this hypothesis, we tested ASO-mediated reduction of *eRF3a* in a hemophilia B mouse model carrying a nonsense mutation. Hemophilia B is an X-linked bleeding disorder that results from a defect in the gene encoding coagulation FIX, a serine protease that is critical for blood clotting [31]. Patients with severe hemophilia B have functional FIX levels that are <1% of normal values and have frequent bleeding episodes that are associated with crippling arthropathy and early death [32]. Increasing circulating FIX to 1% of normal levels can substantially ameliorate the bleeding phenotype [33]. In this hemophilia B mouse model, a human *FIX* minigene, either wild-type (*hFIX-WT* as control) or with a nonsense mutation R338X (*hFIX-R338X*), is expressed in mice that lack the endogenous mouse *FIX* gene [30]. *hFIX-WT* mice contain two copies of the transgene and express hFIX mRNA and protein at similar levels to that of FIX expression in human liver and plasma, respectively. *hFIX-R338X* mice contain six copies of the transgene and express *hFIX* mRNA approximately four-fold higher than human *FIX* mRNA level in liver. Truncated protein expressed from the *hFIX-R338X* transgene is detected in mouse liver but not in plasma. These mice effectively recapitulate the phenotype of patients carrying the R338X mutation (CGA to TGA) demonstrating severe hemophilia with no detectable circulating FIX protein [30]. The *hFIX* transgenes are driven by the human *transthyretin* promoter and thus are primarily expressed in mouse hepatocytes, which are the main cell type for endogenous FIX production [30]. Therefore, it is an optimal model for demonstrating proof-of-concept for ASO-mediated translational read-through approach.

As the *hFIX-R338X* transgene is primarily expressed in hepatocytes, we evaluated whether we could further deplete *eRF3a* in hepatocytes using a triantennary *N*-acetyl galactosamine (GalNAc)-conjugated *eRF3a* ASO to improve the read-through efficacy while reducing any unwanted toxic effects in other cell types. GalNAc is a high-affinity ligand for the hepatocyte-specific asialoglycoprotein receptor (ASGPR) [34]. GalNAc conjugation results in preferential ASO delivery to hepatocytes relative to extrahepatic cells, resulting in 10-fold higher ASO potency in mouse liver than unconjugated ASOs of the same sequence [35]. This enables lower doses of ASO to be administered to reduce hepatocyte targets while largely sparing target reduction in extrahepatic tissues.

**FIG. 2.**
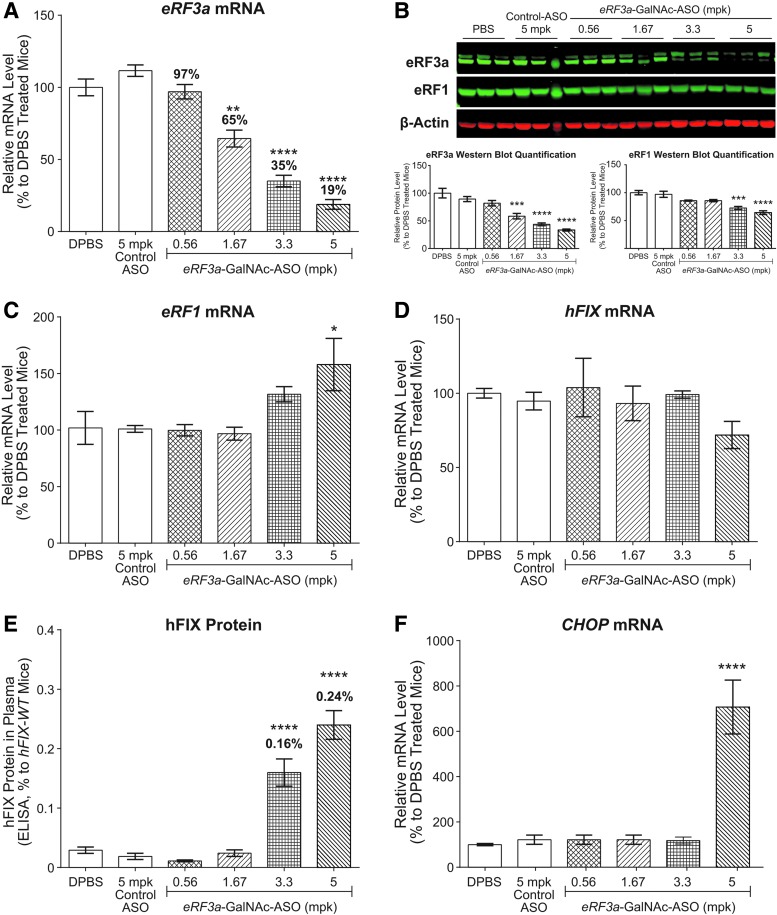
ASO targeting *eRF3a* moderately improves translational read-through in *hFIX-R338X* mice. *hFIX-R338X* male mice 6–12 weeks of age (*n* = 4–6) were treated weekly with four total doses of DPBS, GalNAc-control ASO, and *eRF3a*-GalNAc-ASO at indicated doses. Necropsy was performed 48 h after the last dose of ASO. Results are presented as means ± standard errors. **(A)** qPCR analysis of *eRF3a* mRNA levels in mouse liver samples. **(B)** Western blot analysis of eRF1 and eRF3a protein levels in mouse liver samples. **(C)** qPCR analysis of *eRF1* mRNA levels in mouse liver samples. **(D)** qPCR analysis of *hFIX* mRNA levels in mouse liver samples. **(E)** Mouse plasma hFIX protein levels as measure by ELISA. Plasma hFIX protein level in *hFIX-WT* mice was set as 100%. **(F)** qPCR analysis of *CHOP* mRNA levels in mouse liver samples. For qPCR analysis, *Gapdh* mRNA was used as an endogenous control. The mRNA expression levels in DPBS-treated mouse livers were set as 100%. For western blot analysis, β-actin was used as endogenous control. Statistical significance was determined using a one-way ANOVA and Dunnett's multiple comparison test in Prism. All groups were compared with DPBS-treated group. *P < 0.05; **P < 0.01; ***P < 0.001; ****P < 0.0001. mpk: mg/kg/week; *CHOP*, C/EBP homologous protein; hFIX, human Factor IX; ELISA, enzyme-linked immunosorbent assay; GalNAc, *N*-acetyl galactosamine; WT, wild type.

Dose–response experiments were performed with GalNAc-conjugated *eRF3a* ASO to determine the optimal level of target reduction to achieve a balance between efficacy and safety in hemophilia mice. Male hemophilia mice between ages 6 and 12 weeks were dosed once a week with ASOs at 0.56, 1.67, 3.3 and 5 mg/kg/week for 4 weeks*. eRF3a*-GalNAc-ASO treatments led to dose-dependent liver target reduction in hemophilia mice, reducing *eRF3a* mRNA levels to ∼97%, 65%, 35%, and 19% with respect to the levels of DPBS-treated mice (Fig. 2A). Western blot analysis indicated similar eRF3a target reduction at the protein level (Fig. 2B). In contrast to data previously reported in HEK293 cells whereby siRNA-mediated depletion of *eRF3a* also significantly reduced eRF1 protein levels [25], reduction of eRF3a in hemophilia mouse livers only caused a minor reduction in eRF1 protein levels (Fig. 2B). Of interest, we observed an upregulation of *eRF1* mRNA levels in mouse livers treated with *eRF3a* ASO (Fig. 2C), indicating that a feedback regulation might exist to increase *eRF1* expression in compensation for the loss of *eRF3a* expression.

Without affecting *hFIX-R338X* mRNA levels (Fig. 2D), 3.3 and 5 mg/kg/week *eRF3a*-GalNAc-ASO treatments led to the detection of full-length hFIX protein in mouse plasma, indicating that reduction of *eRF3a* to <35% of normal levels induces translational read-through of *hFIX-R338X* mRNAs (Fig. 2E). However, in contrast to other treatment groups, mice that were treated with *eRF3a*-GalNAc-ASO at 5 mg/kg/week exhibited less body weight gain during the study and slightly elevated liver transaminase levels (data not shown), confirming that reducing *eRF3a* RNA level to <28% of normal levels in liver is not well tolerated in mice (Fig. 1E–H) even with GalNAc-conjugated ASO that restricts target reduction in the liver primarily to hepatocytes [35]. In line with these observations, an upregulation of *CHOP* mRNA was also observed in mice treated with 5 mg/kg/week *eRF3a*-GalNAc-ASO (Fig. 2F), indicating induction of the unfolded protein response (UPR). This effect is likely because of the impairment of global translation accuracy, which is in agreement with a previous report that high concentrations of aminoglycoside treatment cause endoplasmic reticulum stress and activation of the UPR [36].

Therefore, we conclude that a minimum of ∼28% *eRF3a* is needed to avoid effects on global translation. Although *eRF3a*-GalNAc-ASO treatment at a well-tolerated dose significantly increased the production of otherwise undetectable full-length hFIX protein, it did not reach the level required (1% of normal levels) to improve the hemophilia disease phenotype [33].

### ASO targeting *eRF1* moderately improves translational read-through in *hFIX-R338X* mice

**FIG. 3.**
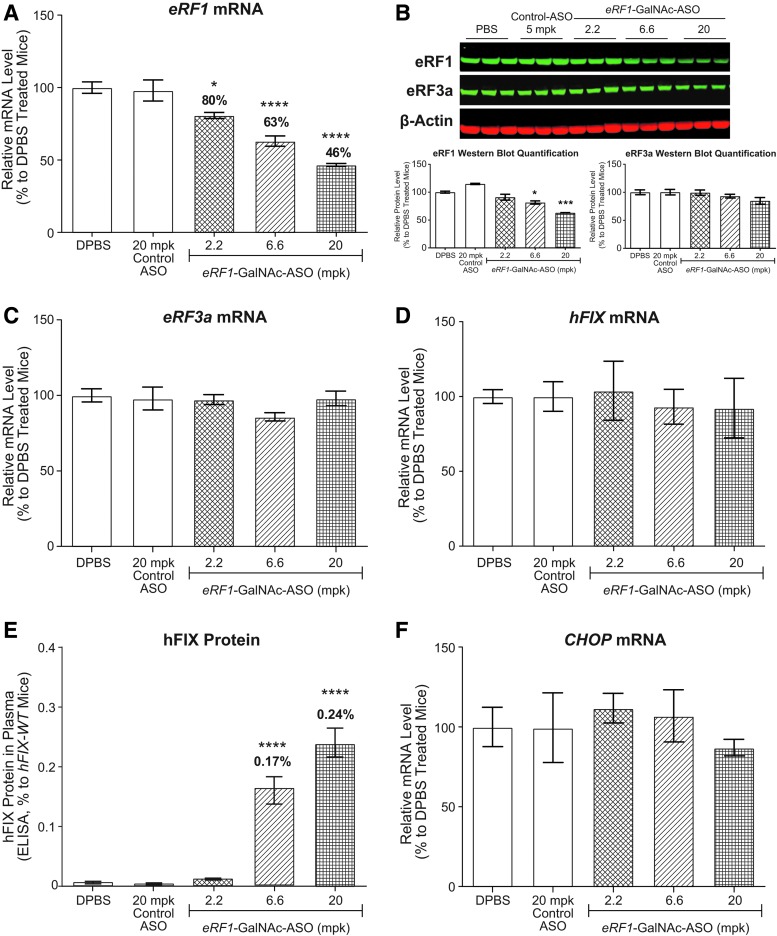
ASO targeting *eRF1* moderately improves translational read-through in *hFIX-R338X* mice. *hFIX-R338X* male mice 6–12 weeks of age (*n* = 4–6) were treated weekly with four total doses of DPBS, GalNAc-control ASO, and *eRF1*-GalNAc-ASO at the indicated doses. Necropsy was performed 48 h after the last dose of ASO. Results are presented as mean ± standard error. **(A)** qPCR analysis of *eRF1* mRNA levels in mouse liver samples. **(B)** Western blot analysis of eRF1 and eRF3a protein levels in mouse liver samples. **(C)** qPCR analysis of *eRF3a* mRNA levels in mouse liver samples. **(D)** qPCR analysis of *hFIX* mRNA levels in mouse liver samples. **(E)** Mouse plasma hFIX protein levels as measured by ELISA. Plasma hFIX protein level in *hFIX-WT* mice was set as 100%. **(F)** qPCR analysis of *CHOP* mRNA levels in mouse liver samples. For qPCR analysis, *Gapdh* mRNA was used as an endogenous control. The mRNA expression levels in DPBS-treated mouse liver were set as 100%. For western blot analysis, β-actin was used as endogenous control. Statistical significance was determined using a one-way ANOVA and Dunnett's multiple comparison test in Prism. All groups were compared with DPBS-treated group. *P < 0.05; ***P < 0.001; ****P < 0.0001.

As reduction of *eRF3a* did not significantly reduce eRF1 protein levels as originally predicted, we evaluated the effects of ASO-mediated reduction of *eRF1* on promoting translational read-through in *hFIX-R338X* hemophilia mice, because it is the translation termination factor that directly recognizes and binds to the stop codon. To deplete *eRF1* more efficiently in hepatocytes, we generated a GalNAc-conjugated *eRF1* ASO. Male hemophilia mice between ages 6 and 12 weeks were dosed once a week with ASOs at 2.2, 6.6 and 20 mg/kg/week for 4 weeks. *eRF1*-GalNAc-ASO treatment led to dose-dependent liver target reduction in hemophilia mice and reduced *eRF1* mRNA levels to ∼80%, 63%, and 46% of DPBS-treated mice (Fig. 3A). Western blot analysis indicated similar eRF1 target reduction at the protein level (Fig. 3B). All treatment groups demonstrated normal body weight gain, and terminal organ weights and blood chemistry, indicating that all treatments were well tolerated (data not shown). Reduction of *eRF1* to these levels did not affect *eRF3a* expression at the mRNA or protein level (Fig. 3B, C).

Although reduction of *eRF1* also did not affect *hFIX-R338X* mRNA levels (Fig. 3D), full-length hFIX protein was detected in plasma of mice treated with *eRF1*-GalNAc-ASO at 6.6 and 20 mg/kg/week, indicating that reduction of *eRF1* to 63% of normal levels induces translational read-through of *hFIX-R338X* mRNA (Fig. 3E). Based on these results, it seems that, in contrast to *eRF3a*, *eRF1* is a haploinsufficient gene in its function in translation termination at PTC. Of interest, we did not detect upregulation of *CHOP* mRNA in any of the treatment groups in this study (Fig. 3F), indicating a lack of UPR induction. These results suggest that ASO-mediated reduction of *eRF1* to 46–63% of normal levels promotes translational read-through at PTC without significantly affecting global translation accuracy.

Therefore, *eRF1* seems to be a safer and preferable target for promoting translational read-through at PTCs in comparison with *eRF3a*. Despite this encouraging data, however, the highest average level of full-length hFIX protein detected in *eRF1*-GalNAc-ASO-treated mice was only ∼0.24% of *hFIX-WT* mice (Fig. 3E), which would not result in meaningful clinical outcomes for hemophilia disease.

### ASOs targeting *eRF1* and *eRF3a* demonstrate synergistic effects with the aminoglycoside geneticin, significantly increasing translational read-through in *hFIX-R338X* mice

Because GalNAc-ASO-mediated reduction of *eRF1* or *eRF3a* demonstrated limited efficacy in promoting translational read-through and seems less likely to be effective as a single therapy, we wanted to test if ASO-mediated reduction of *eRF1* or *eRF3a* will improve the efficacy of the aminoglycoside geneticin (G418), which was previously shown to promote translational read-through in *hFIX-R338X* mice [37]. We treated hemophilia mice with the highest tolerable dose of either *eRF1*-GalNAc-ASO (20 mg/kg/week) or *eRF3a*-GalNAc-ASO (3.3 mg/kg/week) in combination with G418. Male *hFIX-R338X* mice between ages 6 and 12 weeks were dosed once a week with either *eRF1*- or *eRF3a*-GalNAc-ASO for 4 weeks. Seven days before the end of study, ASO-treated cohorts were divided and half were administered 28 mg/kg G418 daily for 7 days [37]. A separate cohort of mice were treated with G418 alone daily for the last 7 days of the study. Animals were sacrificed 48 h after the last ASO treatment and 9 h after the last dose of G418. All groups of animals demonstrated normal body weight gain throughout the study, as well as normal organ weights and plasma transaminase levels as measured at the time of necropsy (data not shown).

**FIG. 4.**
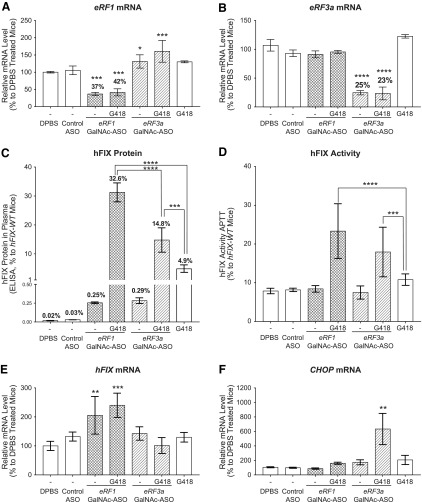
ASOs targeting *eRF1* and *eRF3a* demonstrate synergistic effects with the aminoglycoside geneticin, significantly increasing translational read-through in *hFIX-R338X* mice. *hFIX-R338X* male mice 6–12 weeks of age (*n* = 3) were treated weekly with four total doses of DPBS, GalNAc-control ASO (20 mg/kg/dose), *eRF1*-GalNAc-ASO (20 mg/kg/dose), and *eRF3a*-GalNAc-ASO (3 mg/kg/dose). G418 (28 mg/kg) was administered daily during the final 7 days of the study either alone or in combination with ASO treatments as described. Necropsy was performed 48 h after the last dose of ASO and 9 h after the last dose of geneticin. Results are presented as mean ± standard error. **(A, B)** qPCR analysis of *eRF1* and *eRF3a* mRNA levels in mouse liver samples. **(C)** Mouse plasma hFIX protein levels as measured by ELISA. Plasma hFIX protein level in *hFIX-WT* mice was set as 100%. **(D)** APTT FIX activity assay in plasma from treated mice. FIX activity in *hFIX-WT* mice was set as 100%. **(E, F)** qPCR analysis of *hFIX* and *CHOP* mRNA levels in mouse liver samples. For qPCR analysis, mouse *Gapdh* mRNA was used as endogenous control. The mRNA expression levels in DPBS-treated animals were set as 100%. Statistical significance was determined using a one-way ANOVA and Dunnett's multiple comparison test in Prism. (**A**, **B**, **D** and **E**) All groups were compared with DPBS-treated *hFIX-R338X* group. **(C, D)** Dunnett's multiple comparison test was performed between ASO/G418 combo groups and G418-treated group *P < 0.05; **P < 0.01; ***P < 0.001; ****P < 0.0001. APTT, activated partial thrombo-plastin time.

In this study, both *eRF1*-GalNAc-ASO and *eRF3a*-GalNAc-ASO demonstrated similar efficacy in promoting translational read-through when administered alone without G418 compared with previous studies (hFIX protein levels in the plasma of *eRF1*- and *eRF3a*-GalNAc-ASO treated mice were 0.25% and 0.29% of that of *hFIX-WT* mice, respectively) (Fig. 4A–C). Of interest, when combined with G418 treatment, both *eRF1*- and *eRF3a*-GalNAc-ASO demonstrated synergistic effects with G418 in promoting translational read-through. *eRF3a*-GalNAc-ASO treatment increased G418 activity by ∼three-fold, increasing plasma hFIX protein levels from ∼4.9% to ∼14.8% compared with untreated *hFIX-WT* mice (Fig. 4C). *eRF1*-GalNAc-ASO improved G418 efficacy even further by more than six-fold, reaching ∼32% of untreated *hFIX-WT* levels (Fig. 4C). Furthermore, both *eRF1*-ASO/G418 and *eRF3a*-ASO/G418 treatments significantly improved FIX activity in an activated partial thrombo-plastin time-based coagulation assay (APTT) compared with G418 alone, with *eRF1*-ASO/G418 demonstrating a trend of greater improvement (Fig. 4D). We also observed an approximately two-fold increase in *hFIX-R338X* mRNA levels in *eRF1*-GalNAc-ASO-treated mouse liver, which we did not observe in the previous dose–response study where slightly less *eRF1* target reduction was achieved (Fig. 4E). As a measurement of the UPR, *CHOP* mRNA level did not significantly change in any treatment groups except in the *eRF3a*-GalNAc-ASO/G418-treated group (Fig. 4F), probably because of additive effects of *eRF3a*-GalNAc-ASO and G418 on global translation accuracy.

Overall, in hemophilia mice, GalNAc-conjugated ASOs targeting *eRF1* and *eRF3a* worked in synergy with the small molecular read-through drug geneticin, significantly increasing production of full-length hFIX protein, which results in improved plasma FIX activity. GalNAc-ASO-mediated reduction of *eRF1* seems to be safer and more effective in promoting the translational read-through of *hFIX-R338X* mRNA than reduction of *eRF3a* when combined with G418.

## Discussion

It has been decades since the discovery that aminoglycoside antibiotics can promote translational read-through at PTCs in both yeast and human [38,39]. Despite various efforts to therapeutically develop aminoglycosides and their derivatives for the treatment of diseases with nonsense mutations, none have been approved by the FDA [6–8]. Ataluren, a small molecule promoting translational read-through by targeting the ribosome to promote specific near-cognate tRNA selection [15,40], is in clinical development in United States and approved in European Union Member States for the treatment of Duchene muscular dystrophy caused by a nonsense mutation. However, it has failed to significantly improve respiratory function in cystic fibrosis (CF) patients with nonsense mutations, according to results of a Phase 3 clinical trial that has recently completed. The high unmet medical need for various genetic diseases caused by nonsense mutations calls for novel strategies to promote translational read-through at PTCs.

In this study, we investigated the therapeutic potential of ASO-mediated reduction of the translation termination factors for the treatment of diseases with nonsense mutations. We demonstrated that ASO-mediated reduction of the translation termination factors *eRF1* and *eRF3a* to ∼40% and ∼30% of normal levels in mouse liver, respectively, is well tolerated (Fig. 1). Using a hemophilia B disease mouse model, we demonstrated that reducing *eRF1* to ∼40% and *eRF3a* to ∼30% of normal levels induced translational read-through of *hFIX-R338X* mRNA, leading to the production of an otherwise undetectable full-length hFIX-protein to the level of ∼0.2% of that in *hFIX-WT* mice (Figs. 2 and 3). Moreover, we discovered that although ASO-mediated reduction of *eRF1* or *eRF3a* alone did not produce sufficient hFIX protein to rescue the disease phenotype, the ASO treatments demonstrated synergistic effects with the aminoglycoside geneticin in promoting translational read-through and produced significant amounts of hFIX protein leading to improved plasma FIX activity that is sufficient for rescuing the disease phenotypes in these mice (Fig. 4). These results suggest that although ASO-mediated reduction of the translation termination factors may not be an effective standalone read-through therapy, it could be a potentially effective therapy if utilized to improve the efficacy of small molecule read-through compounds. It will be interesting to test if the read-through improvement observed by ASO-mediated reduction of translation termination factors in combination with G418 also applies to other aminoglycosides or other small molecule read-through drugs such as Ataluren [15,41].

One note of caution is that the incorporation of certain amino acids at a PTC by near-cognate aminoacyl-tRNAs could lead to reduced protein function [42]. Moreover, the identity of the incorporated amino acid is sequence-context dependent [43] and also can be different depending on the small molecule translational read-through reagent used [40]. Therefore, it will be important to characterize the identity of the amino acid inserted by the treatment of ASOs targeting the translation termination factors in an mRNA-specific manner.

We also performed a side-by-side comparison to determine which translation termination factor is a better ASO target to safely induce efficient translational read-through at PTCs. Our results suggest that *eRF1* is a better and more robust target than *eRF3a* for this approach. First, the reduction of *eRF1* to 63% of normal levels can induce translational read-through of *hFIX-R338X* mRNA (Fig. 3), whereas *eRF3a* needs to be depleted to 35% of normal levels to induce translational read-through of the same transcript (Fig. 2). Second, although reduction of *eRF1* and *eRF3a* elicits a similar magnitude of translational read-through without G418, *eRF1* reduction demonstrated much greater activity in improving the efficacy of G418 in generating stable full-length hFIX protein (Fig. 4). Third, although eliciting similar levels of read-through, *eRF1* reduction seems to be better tolerated than *eRF3a* reduction, because the UPR is only triggered upon *eRF3a* depletion as demonstrated by upregulation of *CHOP* mRNA (Fig. 4). Therefore, we conclude that *eRF1* is a better potential therapeutic target to be utilized in combination with small molecule read-through compounds.

It is not clear why *eRF1* reduction seems to promote translational read-through more efficiently compared with *eRF3a* reduction without inducing the UPR. It has been suggested that eRF1 binding to the stop codon is the rate-limiting step for translation termination [44]. It is possible, since translation termination at PTCs is less efficient compared with normal stop codons, that a higher concentration of eRF1 is required for efficient translation termination at PTCs. Therefore, a slight reduction of eRF1 to 63% of normal levels can lead to increased translational read-through at PTCs without significantly impacting normal translation termination efficiency. Of interest, we observed an approximately two-fold increase in *hFIX-R338X* mRNA levels in *eRF1*-GalNAc-ASO-treated mouse liver (Fig. 4D), which probably contributed to the significant elevation of plasma hFIX protein levels in these mice. It remains to be investigated how the reduction of *eRF1* leads to the upregulation of *hFIX* mRNA and whether it affects mRNA stability globally.

We also found that *eRF1* mRNA level is upregulated in *eRF3a*-ASO-treated mice (Fig. 3), indicating that a feedback regulation might exist to ensure efficient translation termination. It was recently demonstrated that expression of *eRF1* in plants is controlled by an autoregulatory circuit involving read-through and nonsense-mediated decay [45]. It will be interesting to determine if this mechanism is conserved in other eukaryotes.

It is surprising to discover that reducing mouse translation termination factors only triggered a minor translational read-through of *hFIX-R338X* mRNA, whereas mutations in *eRF1* and *eRF3a* in yeast induced significant translational read-through [46–48]. These results indicate there might be other proteins that can serve as translation termination factors or additional mechanisms to govern translation termination in higher eukaryotes. Alternatively, reducing *eRF1* and *eRF3a* expression simultaneously might lead to greater translational read-through. More studies will need to be performed to provide further understanding of translation termination at normal and premature stop codons.

The efficiency of translation termination depends on the context of the stop codon and its surrounding sequences [49]. The PTC and its surrounding sequences of *hFIX-R338X* are UGU CUU UGA UCU ACA, a sequence context with high termination efficiency [50]. It was demonstrated that the weakest termination contexts were most affected by increases or decreases in the concentration of eRF1 protein [50]. Therefore, it is possible that in a weaker termination context, ASO-mediated reduction of *eRF1* or *eRF3a* could be effective as a single treatment to induce efficient translational read-through and produce sufficient full-length protein to rescue the disease phenotype.

It was found in a clinical study that the aminoglycoside gentamicin shows variable read-through efficiency in CF patients carrying the W1282X nonsense mutation [51,52]. Further evaluation revealed that the variation in efficacy corresponded with the level of CF transmembrane conductance regulator (*CFTR*) mRNA, leading to the discovery that the efficiency of NMD might vary in these patients and hence affect the response to read-through treatments [53,54]. We previously demonstrated that inhibition of NMD by ASO-mediated reduction of a branch-specific NMD factor, Upf3b [55,56], significantly stabilized *hFIX* mRNA harboring an early nonsense mutation (R29X) and significantly improved the efficacy of read-through therapy in this mouse hemophilia model [57]. These results underlined the need for a safe and effective read-through therapy and suggested that a combination strategy to simultaneously modulate both the NMD pathway and translation termination process is required to treat genetic diseases caused by nonsense mutations. Overall, our data indicate that modulating the translation termination pathway by ASOs may provide a novel therapeutic approach for human genetic diseases that are caused by nonsense mutations.
